# Mineral and Phytochemical Profiles and Antioxidant Activity of Herbal Material from Two Temperate* Astragalus* Species

**DOI:** 10.1155/2018/6318630

**Published:** 2018-01-21

**Authors:** Bronislava Butkutė, Audronė Dagilytė, Raimondas Benetis, Audrius Padarauskas, Jurgita Cesevičienė, Vilma Olšauskaitė, Nijolė Lemežienė

**Affiliations:** ^1^Chemical Research Laboratory, Institute of Agriculture, Lithuanian Research Centre for Agriculture and Forestry, Instituto Al. 1, Akademija, LT-58344 Kėdainiai, Lithuania; ^2^Department of Drug Chemistry, Faculty of Pharmacy, Lithuanian University of Health Sciences, A. Mickevičiaus 9, LT-44307 Kaunas, Lithuania; ^3^Department of Analytical and Environmental Chemistry, Vilnius University, Naugarduko 24, LT-03225 Vilnius, Lithuania; ^4^Department of Grass Breeding, Institute of Agriculture, Lithuanian Research Centre for Agriculture and Forestry, Instituto Al. 1, Akademija, LT-58344 Kėdainiai, Lithuania

## Abstract

Only a few species of the large* Astragalus* genus, widely used for medicinal purposes, have been thoroughly studied for phytochemical composition. The aim of our research was to investigate the rarely studied species* A. glycyphyllos* L. and* A. cicer* L. for the distribution of mineral elements and phytochemicals in whole plants at two growth stages and in morphological fractions. We also investigated the capacity of the plant extracts to scavenge 2,2-diphenyl-1-picrylhydrazyl (DPPH) radicals and to chelate ferrous ions. Chemical composition and antioxidant properties depended on species, maturity, and plant part. Herbal material of* A. glycyphyllos* was richer in Fe, total phenolics, and flavonoids, whereas extracts of* A. cicer* showed a higher antioxidant activity. Young plants had more isoflavones, showed greater quenching of DPPH radicals, and exhibited better mineral profiles than flowering plants. Among plant parts, leaves were the most valuable plant material according to most characteristics investigated. Isoflavone concentration in flowers was lower than in leaves and stems. None of the* Astragalus *samples contained detectable amounts of the alkaloid swainsonine. The study demonstrates the potential of plant material from two* Astragalus* species as a valuable source of iron, phenolic substances including isoflavones, free-radical scavengers, and Fe^2+^ chelators for pharmaceutical use.

## 1. Introduction

During the past few years, a revival of interest in botanical sources of natural drugs, cosmeceuticals, nutraceuticals, herbal teas, and other health-promoting products has been appreciable. The medicinal use of* Astragalus* species dates back to more than 2000 years ago [1]. A wide range of medicinal applications of remedial preparations have been demonstrated and described in many publications [2–4]. Researchers have demonstrated their immunostimulant activity, antiviral effects, and immunorestorative function in various cancers. Particular attention from the viewpoint of both medicinal use and scientific investigation has been and remains focused on several species of* Astragalus *genus, the largest one among flowering plants, which is comprised of about 3000 species [5]. Species* A*.* membranaceus* (Fisch.) Bge. and* A. membranaceus* var.* mongolicus *(Bge.) Hsiao are important herbs in traditional Chinese medicine. These species are indigenous to China, Korea, Mongolia, and Siberia and are commercially cultivated in northern China and Korea; their herbal material has been studied in detail for chemical composition and pharmacological applications [3, 4, 6–8].

The two* Astragalus* species chosen for this study grow naturally in the temperate climatic conditions of Eurasia.* Astragalus cicer *L. (cicer milkvetch) is a perennial plant native to Central and Eastern Europe [9], which was introduced to areas in Southern Europe, North America, and South America.* Astragalus glycyphyllos *L., commonly known as liquorice milkvetch, is also a perennial species which is widespread throughout Europe and temperate Asia [10]. These two species have been used in traditional medicine as well as in food in several European countries [7, 11]. Application of remedies from roots and leaves of* A. glycyphyllos *relates to their refreshing, purifying, diuretic, and many other properties [7, 8, 12, 13]. Lysiuk et al. [14] quantified hydroxycinnamic acids in the plant material of* A. glycyphyllos* and stated that some of them might contribute to a positive impact on the urinary system due to their renoprotective effects. According to De Vos [15], the use of cicer milkvetch as a medicinal herb was listed in eight sources, beginning with the Hippocratic Corpus of the 5th century BC and ending with the Farmacopea Española of 1865. Although cicer and liquorice milkvetches have been in use for a long period of time, there are few data available on their chemical composition and bioactive properties.

Qualitative data on the occurrence of flavonoids in both above- and underground parts of* Astragalus* species were summarised in recent review articles [2, 16, 17]. Based on reviews, it appears that flavonoid-like compounds identified in* A. cicer* and* A. glycyphyllos* are species-specific. This species specificity may be related to the taxonomic differences between species. Based on phylogenetic analyses, species were attributed to the different taxonomic units [18–20].

There are numerous reports on the association between phytoestrogens and reduced osteoporosis and cardiovascular disease, prevention of cancer, antidiabetic effects, and relief of menopausal symptoms [21]. Li et al. [2] confirmed that several isoflavones and their derivatives were detected in* Astragalus *spp., including formononetin, biochanin A, and genistein. However, these data were related to species not included in this study. The information on isoflavone distribution in plant material of* A. glycyphyllos* and* A. cicer* is scarce and generally limited to qualitative data [16, 17]. In addition to valuable bioactive compounds and properties,* Astragalus* species contain toxic substances, including the neurotoxin indolizidine alkaloid, swainsonine [8, 22].

Phenolic substances are major contributors to the antioxidant properties of plants, acting as free-radical scavengers and chain breakers [23]. Despite that, iron deficiency results in impaired production of iron-containing proteins and inhibition of cell growth; excessive iron uptake has been related to hereditary hemochromatosis, leading to tissue damage [24]. According to Santos et al. [25], iron chelation may be useful in the prevention and treatment of microbial infections. However, we did not find published data on ferrous ion-chelating ability by extracts of* A. cicer* and* A. glycyphyllos* plants.

Diets of over two-thirds of the world's population lack one or more essential mineral elements [26]. The minerals, iron (Fe), zinc (Zn), calcium (Ca), potassium (K), and magnesium (Mg), are classified as components of high priority for dietary supplementation [27]. Currently, Fe and Zn are the most common microelements lacking in diets of young children and women of childbearing age, particularly in low- and middle-income countries [28]. Investigations of* Astragalus cicer* and* A. glycyphyllos* with respect to mineral and phytochemical compositions, as well as antioxidant activity, remain sporadic and mostly of a qualitative nature.

In this study, we investigated the distribution of minerals and bioactive substances in the aerial parts of two European* Astragalus* species and conducted an* in vitro *evaluation of antioxidant activity in plant extracts. The influence of growth stage and plant part on these parameters was examined.

## 2. Materials and Methods

### 2.1. Plant Materials

**Figure 1:**
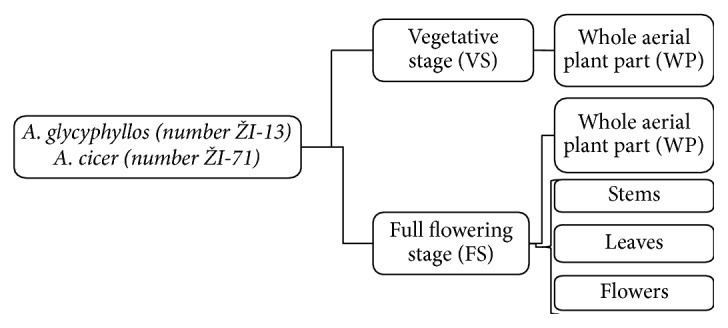
Sampling design.

Five samples of each of two* Astragalus* species (*A. cicer* L. and* A. glycyphyllos* L.) were used in the current investigation (Figure 1, Supplementary Materials). Seeds of wild ecotypes of* A. glycyphyllos* and* A. cicer* were collected in natural habitats in Lithuania (55°22′51′′N; 23°50′35′′E) and Latvia (57°01′45′′N; 21°25′23′′E), respectively. Species were identified by Dr. Vaclovas Stukonis (Department of Grass Breeding, Institute of Agriculture, Lithuanian Research Centre for Agriculture and Forestry, IA, LAMMC) according to the morphological descriptions [19, 29]. Species were catalogued at IA, LAMMC, under the respective numbers ŽI-13 and ŽI-71.

The following year, seeds were sown in the germplasm collection of the perennial legumes species in single rows, 2.5 m long and 0.5 m apart. Plots were set up in the experimental site of IA, LAMMC (Central Lowland of Lithuania, 55°23′49′′N, 23°51′40′′E). A randomised complete block design was used with four replications. No pesticides were applied. Samples of milkvetch species were collected during the fully flowering and vegetative stages. Aerial parts of each legume sampled at full flowering were divided into two subsamples: one was investigated as a sample of the whole aerial part; the other subsample was divided into three plant parts (stems, leaves, and flowers) (Figure 1).

The four replicate samples were pooled for chemical analyses. All samples were washed thoroughly with tap water, rinsed with distilled water, and blotted on filter paper. Samples were then chopped, immediately predried in an oven (105°C for 15 min) to rapidly stop the processes of metabolism, oven-dried (65 ± 5°C for 24 h), and then ground using a cyclone mill and passed through a 1 mm screen. Before analysis, a small portion (2-3 g) of each sample was dried to a constant mass in a forced-air oven at 105 ± 5°C so that data could be expressed per unit dry matter (DM). Chemical analyses were carried out in triplicate.

### 2.2. Analytical Standards and Reagents

Daidzein (7-hydroxy-3-(4-hydroxyphenyl)-4H-chromen-4-one; ≥98%), formononetin (7-hydroxy-3-(4-methoxyphenyl)-4H-chromen-4-one; ≥99%), genistein (5,7-dihydroxy-3-(4-hydroxyphenyl)-4H-chromen-4-one; ≥98%), biochanin A (5,7-dihydroxy-3-(4-methoxyphenyl)-4H-chromen-4-one; ≥98%), swainsonine (≥99%), Folin-Ciocalteu phenol reagent (2 N), gallic acid monohydrate (≥98.0%), sodium carbonate (anhydrous, 99.5–100%), 3-(2-pyridyl)-5,6-diphenyl-1,2,4-triazine-4′,4′′-disulfonic acid monosodium salt (ferrozine; 97%), methanol (≥99.9%), acetone (≥99.8%), hexane (≥97.0%), acetic acid (≥99.7%), sulphuric acid (95–98%), LC-MS grade acetonitrile, formic acid, acetic acid, ammonium formate, and ammonium acetate were purchased from Sigma-Aldrich (UAB Labochema, Vilnius, Lithuania). LC-MS grade methanol, acetic acid, and formic acid were obtained from Fluka/Sigma-Aldrich (UAB Labochema). Stable free DPPH (2,2-diphenyl-1-picrylhydrazyl) radical (95%) and iron(II) chloride (anhydrous, 99.5%) were supplied by Alfa Aesar GmbH & Co. KG (Karlsruhe, Germany). Acetic acid (100%), aluminium chloride hexahydrate (≥95%), hexamethylenetetramine (≥99%), and rutin trihydrate (≥95%) were obtained from Carl Roth GmbH + Co. KG (Karlsruhe, Germany). Ethanol (96.3% v/v) was purchased from Stumbras (Kaunas, Lithuania). Double-deionised water with conductivity lower than 18.2 M*Ω* was purified using a Milli-Q Direct 8 water purification system (Millipore, Bedford, MA, USA).

### 2.3. Determination of Minerals

Concentrations of potassium, calcium, magnesium, zinc, and iron were quantified after nitric acid plus hydrogen peroxide digestion followed by flame atomic absorption spectroscopy (AAS) using a PerkinElmer model AAnalyst 200 (USA). Parameters of the instrument were chosen in accordance with the manufacturer's instructions. Total phosphorus was determined after sulphuric acid digestion of the samples and reaction with molybdate vanadate. The absorbance was measured by a UV-Vis spectrophotometer (Cary 50, Varian, USA) at 430 nm. Mineral content was expressed as mg/100 g DM.

### 2.4. Preparation of the Extracts

Preliminary extraction studies were performed to determine the effect of the extraction method (maceration and ultrasonic agitation) and aqueous ethanol (40–80% v/v) on the recovery of total phenolic contents (data not shown). It was established that the highest recovery of phenolics was achieved using 70% (v/v) aqueous ethanol and ultrasonic extraction of botanical samples at 50°C for a 15-minute period of sonication. An ultrasonic bath Elmasonic S40H (Elma Schmidbauer GmbH, Germany) was used for sonication extractions. Samples (0.25 g) of powdered (oven-dried) plant material plus 25 mL of 70% (v/v) aqueous ethanol were sonicated at 50°C for 15 min. The suspension was filtered and the supernatant adjusted to 25 mL in a measuring flask.

Acid hydrolysis and extraction of isoflavones were both performed in a single step according to a slightly modified procedure described by Saviranta et al. [30]. The representative amount of samples (250 mg) was extracted with 10 mL of methanol/water (8 : 2, v/v) containing 2 M HCl using sonication for 30 min at room temperature before being hydrolysed at 80–85°C for 1.5 h. Extracts were filtered through a 0.2 *μ*m nylon syringe filter and then analysed.

Swainsonine extraction was performed according to Gardner and Cook's [31] published procedure. Dried plant material (100 mg) was placed in a 10 mL screw-cap glass container and extracted with 5 mL of 2% acetic acid for 16 h with agitation. After extraction, the samples were centrifuged for 5 min. Aliquots (0.50 mL) of the extract were added to 1.00 mL of acetonitrile, thoroughly mixed, filtered through a 0.2 *μ*m nylon syringe filter into a glass sample vial, and then analysed.

### 2.5. Total Phenolic Content

Total phenolic content (TPC) of plant extracts was determined spectrophotometrically by the Folin-Ciocalteu method [32]. Standard solutions of gallic acid were prepared at concentrations of 11–350 *μ*g/mL in ethanol (96% v/v). Aliquots (1 mL) of the standards or appropriately diluted extract were combined with 0.2 N Folin-Ciocalteu's reagent (5 mL). After 5 min, sodium carbonate (4 mL of 7.5% w/v solution) was added to the mixture and shaken. Samples were left to stand for 60 min at room temperature and then absorbance was determined at 765 nm using a UV-Vis spectrophotometer, Spectronic Genesys 2 (Spectronic Instruments, USA). Quantification was based on the gallic acid standard curve. TPC concentration was expressed in mg of gallic acid equivalents (GAE) per g DM (mg GAE/g).

### 2.6. Total Flavonoid Content

Analysis of extracts for total flavonoid content (TFC) was performed by spectrophotometry, as described in the Lithuanian Pharmacopoeia [33]. A 1 mL aliquot of plant extract (Section 2.4) was added to a 25 mL volumetric flask containing 10 mL of 96% (v/v) ethanol. Then, 0.5 mL of 33% acetic acid, 1.5 mL 10% AlCl_3_, and 2 mL of 5% hexamethylenetetramine solution were pipetted into the flask and made up to 25 mL with distilled water. Absorbance was read at 407 nm after 30 min at 20°C versus the prepared blank. Blank samples were prepared from 1 mL of plant extract, 10 mL of 96% (v/v) ethanol, and 0.5 mL of 33% acetic acid and diluted to 25 mL with distilled water. The absorbance of the reference solution, which was prepared using 1 mL of rutin solution instead of plant extract, was measured simultaneously. Standard rutin solution was prepared by dissolving 0.05 g of rutin in 100 mL of 96% ethanol. TFC was expressed as milligrams of rutin equivalents (RE) per g on DM (mg RE/g).

### 2.7. Quantification of Isoflavones

Quantification of the four isoflavones (daidzein, genistein, and their 4′-methylated derivatives, formononetin and biochanin A) was performed by ultraperformance liquid chromatography (UPLC) using a Waters Acquity UPLC system (Waters, Milford, MA, USA) equipped with a binary pump, membrane degasser, autosampler, thermostated column compartment, and diode array detector (DAD). An Acquity UPLC BEH C18 column (100 × 2.1 mm i.d., 1.7 *μ*m, Waters) was used in the experiments. Elution was performed using 0.25% aqueous acetic acid (mobile phase A) and 0.25% acetic acid in 80 : 20 v/v methanol/water (mobile phase B). The flow rate was 0.25 mL/min with a linear gradient from 2% to 100% B in 15 min, followed by reequilibration with the initial mobile phase for 5 min. Column temperature was maintained at 30°C, the mobile phase flow rate was 0.25 mL/min, and the injection volume was 5 *μ*L. Data collection and management were performed using HyStar 3.2 software (Bruker Daltonics, Bremen, Germany). Isoflavones in extracts were identified according to our recently published procedure [34] and by comparing retention times with those of corresponding standards. Quantification was performed by external calibration and results are expressed as mg/100 g DM. The limits of quantification, defined as the concentration resulting in a signal ten times the noise level, were 0.15 mg/L (0.006 mg/g DM) for biochanin A and formononetin, 0.20 mg/L (0.008 mg/g DM) for genistein, and 0.25 mg/L (0.010 mg/g DM) for daidzein.

### 2.8. LC-MS/MS Conditions of Swainsonine Detection

Hydrophilic interaction chromatography (HILIC) combined with tandem mass spectrometry (MS/MS) was used for quantification of swainsonine [35]. All separation procedures were carried out using a 1290 Infinity UHPLC system connected to 6410 triple quadrupole mass spectrometer, equipped with electrospray ionisation (ESI) source (Agilent Technologies, USA). HILIC separation was performed on an Acquity UPLC BEH HILIC column (2.1 × 100 mm, 1.7 *μ*m, Waters). The first and third quadrupoles were operated at unit resolution. Data were acquired and processed using MassHunter software (Agilent).

### 2.9. DPPH Radical-Scavenging Activity

The antioxidant activity of plant extracts was determined based on free-radical scavenging capacity using the stable 2,2-diphenyl-1-picrylhydrazyl (DPPH) radical [36]. A solution of DPPH in 96% (v/v) ethanol (6 × 10^−5^ M or 0.06 *μ*moles of pure DPPH per 1 mL solution) was prepared daily before analysis. 50 *μ*L of the plant extract (Section 2.4) was mixed with 2 mL of DPPH solution and left to stand for 30 min (until the reaction reached a steady state) in the dark at room temperature. The decrease in absorbance due to scavenging of DPPH was monitored with a spectrophotometer at 515 nm. The absorption of a blank sample containing the same amount of 70% (v/v) ethanol and DPPH solution was determined each time before the analysis. The radical-scavenging capacity of plant extracts was calculated as the percentage of DPPH inhibition (DPPH%) according to the following equation:

(1) $$\begin{array}{c} DPPH\%=\frac{\left[\left(A_{control}-A_{sample}\right)\times 100\%\right]}{A_{control}}, \end{array}$$

where *A*_control_ is the absorption of the blank sample (*t* = 0 min) and *A*_sample_ is the absorption of the solution containing the plant extract (*t* = 30 min). Finally, results were recalculated as *μ*moles of DPPH free radicals scavenged by the extract from 1 g of plant material DM (DPPH *μ*mol/g) according to the following equation:

(2) $$\begin{array}{c} DPPH\;\;\mu mol/g=\frac{\left(0.12\times DPPH\%\right)}{\left(0.0005\times 100\right)}, \end{array}$$

where 0.12 is *μ*moles of pure DPPH in the aliquot and 0.0005 is the plant material mass (g) in the volume of extract used for the test.

### 2.10. Ferrous Ion-Chelating Activity

Ferrous ion-chelating (FIC) potential of the extract was investigated according to the modified method of Dinis et al. [37], wherein the Fe2^+^-chelating ability was determined by measuring ferrous iron-ferrozine complex at 562 nm. Briefly, 50 *μ*L of 2 mM FeCl_2_ solution was added to a 1 mL aliquot of 70% (v/v) ethanolic plant extract (appropriately diluted). After 5 min, the reaction was initiated by the addition of 0.2 mL of 5 mM ferrozine solution. The mixture was shaken vigorously and left to stand at room temperature for 10 min. The absorbance of the solution was measured at 562 nm. A 1 mL aliquot of 70% (v/v) ethanol, containing no plant extract, was used as the control. The percentage of ferrous ion-chelating activity (FIC%) of the extracts was calculated according to the following equation:

(3) $$\begin{array}{c} FIC\%=\frac{\left[\left(A_{control}-A_{sample}\right)\times 100\%\right]}{A_{control}}, \end{array}$$

where *A*_control_ is the absorbance of the control and *A*_sample_ is the absorbance of the reaction mixture containing the plant extract.

The final FIC capacity was calculated as the amount of Fe^2+^*μ*moles bound by chelating agents in the extract from 1 g of plant material DM (FIC *μ*mol/g) according to the following equation:

(4) $$\begin{array}{c} FIC\;\;\mu mol/g=\frac{\left(0.1\times FIC\%\right)}{\left(0.01\times 100\right)}, \end{array}$$

where 0.1 is Fe^2+^*μ*moles in the aliquot of FeCl_2_ solution and 0.01 is the plant material mass (g) equivalent to the volume of extract used for the test (1 mL).

### 2.11. Statistical Analysis

Statistical analysis of data was carried out using Statistica 7.0 software for Windows (StatSoft Inc., USA), performing basic statistics and correlation matrices. Results for mineral composition, bioactive compound, and antioxidant activity were presented as the mean of triplicate determinations ± standard deviations (SD) and expressed on a DM basis. The Pearson correlation coefficient test was performed on values obtained for isoflavones, TPC, TFC, DPPH, and FIC.

## 3. Results and Discussion

### 3.1. Mineral Composition

The mineral concentration of* Astragalus* spp. varied widely depending on the plant growth stage and morphological fraction (Table 1). In general, the mineral profile of young plants demonstrated higher content of ash and almost all elements tested than the flowering milkvetches. With respect to the growth stage, Fe concentration changed the most out of all the mineral components, decreasing as much as 3-fold in* A. glycyphyllos *and 2-fold in* A. cicer* with advancing maturity: plants at the vegetative stage contained 65.29 and 30.19 mg Fe/100 g, respectively, whereas Fe concentration in flowering plants amounted to 21.72 and 14.94 mg/100 g, respectively. Within the species, the mineral concentrations (except for Fe) differed more among plant parts (from 1.6- to 4.1-fold) than between the two growth stages (by 1- to 1.5-fold). In flowering* Astragalus* plants, the content of ash, Ca, Mg, and Fe was higher in leaves than in flowers and stems, and flowers were richer in K, P, and Zn than in the remaining two morphological fractions. The mineral profile of stems was the poorest among all samples of respective species of milkvetch; however, with regard to Fe concentration, stems of* A. glycyphyllos *were Fe-richer (15.85 mg/100 g) than its flowers (14.1 mg/100 g).

The growth stage at harvest as the most important factor governing the quality of various herbaceous plants is well documented in the scientific literature. Data on this subject for the species studied here were not available; however, research results on other perennial legumes demonstrated that harvest at an early phenological stage is preferred with regard to higher concentration of some macro- and micronutrients [38, 39]. Changes in mineral content with advancing plant maturity are related primarily not only to the increasing ratio of stem with low mineral content to leaf distinguished by higher concentration of elements, but also to a mineral dilution effect caused by accumulation of lignocellulosic components in mature plants. Results on the distribution of minerals among plant parts reported here are in agreement with earlier observations [38, 39] for other genera of Fabaceae.

With regard to the mineral profile,* Astragalus* species, in particular young milkvetch plants harvested at the vegetative stage and leaves of fully flowering plants, are a good source of important dietary elements such as Ca, Mg, and Fe. Additionally, it should be noted that Fe values for young* Astragalus* plants were higher than those in other plants widely used in foods, including spinach which is considered to be a rich source of Fe, or in many medicinal plants [26, 40]. This finding is significant as Fe deficiency is considered to be one of the top ten health challenges in modern society, being particularly prevalent in women of childbearing age [41]. Findings of this study indicate that* Astragalus* plant material could provide an important natural source of iron.

### 3.2. Bioactive Substances

**Figure 2:**
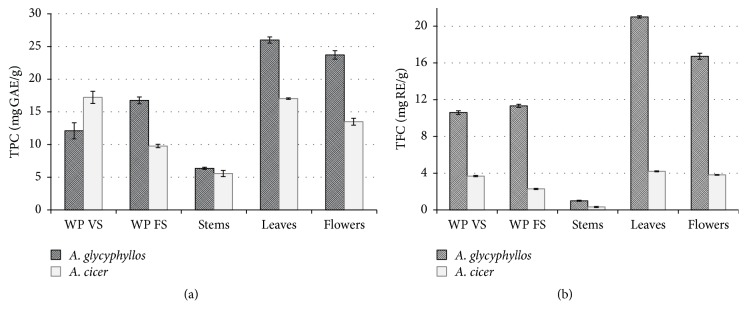
Total phenol (a) and total flavonoid (b) content in* A. glycyphyllos* and* A. cicer* plant material: the whole aerial parts (WP) of plants of vegetative stage (VS) and flowering stage (FS) and the separate parts of fully flowering plants (error bars indicate standard deviation).

Extracts of* A. glycyphyllos *leaves and flowers had the greatest amount of phenolic compounds (25.99 and 23.71 mg GAE/g, resp.; Figure 2(a)) and total flavonoids (21.00 and 16.71 mg RE/g, resp., Figure 2(b)). The lowest concentrations of both total phenolics (6.36 mg GAE/g) and flavonoids (1.00 mg RE/g) were measured in stems of flowering liquorice milkvetch plants. The same trend was observed with regard to TPC and TFC distribution in plant parts of flowering* A. cicer*; however, concentrations of both total phenolics and flavonoids were considerably lower than those in the respective plant material of* A. glycyphyllos*. Differences in TPC and TFC concentrations in whole aerial plant parts were observed between the two stages of plant growth, with concentrations being lower in young plants of* A. glycyphyllos* than in fully flowering plants and vice versa in* A. cicer*.

Overall, results for TPC and TFC are consistent with research data presented in the literature, although published data are very sparse for the plant species studied here. Platikanov et al. [18] demonstrated that composition of volatile compounds, including those of phenolic origin, varied according to plant part of the various* Astragalus* spp. plants and within the vegetation period. Lobanova [42] similarly showed that, at the beginning of the vegetation period,* A. glycyphyllos* leaves contained fewer flavonoids (flavonols) than leaves at later stages of maturity (flowering and beginning of fruiting). Our data agree with this finding, suggesting that, among plant parts, stems accumulate the lowest concentrations of flavonoids. However,* A. glycyphyllos* collected in the mountains of Serbia and Montenegro accumulated considerably more TPC [12] than we found in herbal material from Lithuanian plants. Meanwhile, Tusevski et al. [43] reported a TPC value (15.93 mg GAE/g) in liquorice milkvetch from Macedonia similar to that found in this study in whole fully flowering plants. However, the Macedonian researchers reported lower TFC (1.62 mg catechin equivalent/g) than we determined in the whole aerial part of plants fully flowering or in plants during the vegetative phase (10.60 and 11.33 mg RE/g, resp.). Since there is a sparsity of published data on this topic, it is difficult to make a definitive statement on the discrepancies described. With regard to TPC and TFC in herbal material from* A. cicer*, published data were not found.

In the current study, we focused on the four major isoflavones (formononetin, biochanin A, genistein, and daidzein) occurring in red clover, a perennial legume species most frequently studied for isoflavones. A further reason for selecting these isoflavones was that daidzein and genistein are the primary isoflavones in soy beans, commonly used as a source of isoflavones in the production of food supplements and functional foods. Numerous differences in both the qualitative and the quantitative patterns of isoflavone composition were observed in herbal material from the two* Astragalus *species (Table 2). The dominant isoflavones in young plants of both* A. glycyphyllos* and* A. cicer *were formononetin (9.24 and 10.85 mg/100 g, resp.) and biochanin A (8.81 and 11.40 mg/100 g, resp.). Concentrations of these isoflavones were considerably lower in the whole aerial part as well as in separate morphological fractions of fully flowering plants, compared to those at the vegetative stage. In contrast, with regard to genistein concentration, the opposite trend was observed: flowering plants and their parts contained more isoflavones than plants in the vegetative phase. Among the plant parts of* A. glycyphyllos*, the highest genistein concentration was found in stems (7.29 mg/100 g). In leaves of flowering* A. cicer*, genistein concentration was of a similar level to that found in stems and the whole aerial plant. There were only trace levels of daidzein (below the limit of quantitation) in extracts of all samples from the two* Astragalus* species. According to the total amount of isoflavones quantified, young plants of* A. cicer *contained more phytoestrogens than those of* A. glycyphyllos*; however, samples of flowering* A. glycyphyllos *were richer in isoflavones than the respective plant part of cicer milkvetch. Isoflavones concentration in flowers of milkvetches was from 1.6- to 2.2-fold lower than in stems and leaves.

**Figure 3:**
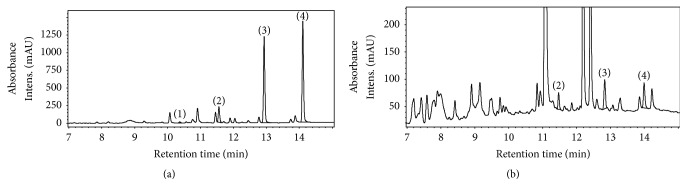
UPLC-UV chromatograms of isoflavones in the extracts of the whole aerial parts of flowering* Trifolium pratense* (a) and* Astragalus glycyphyllos* (b). Peaks: (1) daidzein, (2) genistein, (3) formononetin, and (4) biochanin A. Chromatographic conditions are described under Materials and Methods.

Comparison of the isoflavone content of milkvetches with that of more than 240 foods, reported by Kuhnle et al. [44], made it evident that only soy-based flour (124.4 mg/100 g) was richer in isoflavones than plant material of* Astragalus *accessions. Moreover, qualitative analysis of UPLC-UV chromatograms of extracts from liquorice milkvetch (Figure 3) showed that the species contains compounds which eluted between daidzein and genistein as well as between genistein and formononetin.

There is evidence to suggest that* Astragalus* species have more bioactive compounds related to isoflavones than the four isoflavones selected for quantification in this study. Among the perennial legume species, isoflavones have only been quantified in some detail in red clover [30, 45, 46], and to a much lesser extent in other* Trifolium* spp. [47, 48] and* Medicago *spp. [49]. Reliable quantitative data on isoflavones in* A. cicer *and* A. glycyphyllos* are currently not available; only sporadic qualitative information can be found in the published literature [16].

The HILIC-MS/MS method was optimised and used to determine swainsonine in two samples of milkvetch species growing in Lithuania. Investigations revealed that neither of the* Astragalus *samples contained detectable amounts of the alkaloid. This finding may be attributed to swainsonine production in* Astragalus* spp. and other allied legume species being strongly correlated with the presence of a fungal endophyte,* Embellisia* spp. [50, 51]. In our study, samples were fungus-free and, consequently, swainsonine was not detected.

### 3.3. Antioxidant Activity

**Figure 4:**
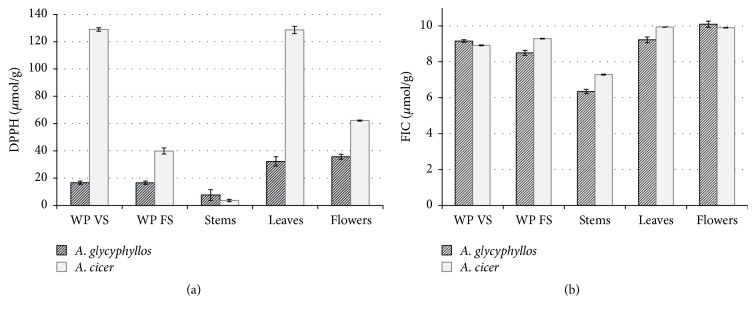
Antioxidant activity: DPPH radical scavenging (a) and ferrous ion-chelating (FIC) capacity (b) of extracts of* A. glycyphyllos* and* A. cicer* plant material: whole aerial parts (WP) of plants of vegetative stage (VS) and fully flowering stage (FS) and the separate parts of fully flowering plants (error bars indicate standard deviation).

The capacity of extracts of* A. glycyphyllos *herbal material to scavenge DPPH free radicals ranged from 7.52 to 35.64 *μ*mol/g (Figure 4(a)). The highest antioxidant activity was observed in extracts from flowers (35.64 *μ*mol/g), followed by leaves (32.26 *μ*mol/g) and the whole aerial part of young plants (16.65 *μ*mol/g). The stems showed the lowest DPPH scavenging activity (7.52 *μ*mol/g). Herbal material of* A. cicer* exhibited a higher potential to scavenge DPPH radicals than respective plant parts of* A. glycyphyllos*, with the exception of stems. This finding was despite the inverse trend in TPC and TFC distribution in the two species (Figures 2 and 4). This observation could be explained by the presence of other bioactive compounds, distinguished by strong free-radical quenching, which were not identified in the current study. It is likely that these compounds would be largely specific to* A. cicer* herbal material. At an early growth stage,* A. cicer* plants exhibited a particularly high antioxidant activity (129.0 *μ*mol/g) similar to that in leaves (128.6 *μ*mol/g). Extracts of flowers showed twice as low DPPH quenching capacity as leaves and young plants, but it was still fairly high (62.17 *μ*mol/g), while stems exhibited negligible free-radical scavenging activity. In relation to the growth stage, plants harvested during the vegetative growth stage demonstrated a higher capacity to scavenge DPPH free radicals than those harvested at flowering.

Extracts from the examined herbal material of the* Astragalus* species showed a high capacity to chelate ferrous ions; Fe^2+^ chelators from 1 g of sample were shown to bind from 6.34 to 10.09 *μ*moles of ferrous ions (Figure 4(b)). Among plant parts of flowering* A. glycyphyllos,* flowers showed the highest FIC capacity (10.09 *μ*mol/g) and stems had the lowest value (6.34 *μ*mol/g). For extracts from leaves and flowers of* A. cicer*, similarly high FIC capacity (9.94 and 9.90 *μ*mol/g, resp.) was characteristic. Regarding changes in FIC values in plants during the two growth stages, a different trend was revealed for each of the species.

Low DPPH radical-scavenging activity of* A. glycyphyllos* extract was also reported by Tusevski et al. [43]. Data on both DPPH scavenging and FIC activities of extracts from* A. cicer* plant material, as well as on FIC capacity of* A. glycyphyllos*, were not found in the published literature. The high FIC capacity of extracts from* Astragalus *species may prove to be of therapeutic importance, since synthetic compounds currently used in chelation therapy have certain side effects [52].

### 3.4. Relationships between Bioactive Substances and Antioxidant Properties

Analysis of linear relationships between values for FIC capacity, DPPH radical-scavenging activity, and phenolic compounds (TPC and TFC) was performed for sample sets of individual* Astragalus* species and the set which included all samples of both species (Table 3).

Generally, the results revealed significant positive correlations between TPC and TFC in all three sample sets. Significant positive linear correlations were established between TPC and DPPH in sample sets from the separate plant species only:* A. glycyphyllos *(*r* = 0.952; *p* < 0.05) and* A. cicer* (*r* = 0.977; *p* < 0.01); however, the relationship between TPC and FIC in sample sets from the separate plant species showed a weaker correlation. Correlations which ranged from weakly negative with insignificant difference (*p* > 0.05) to strongly positive were observed between TFC and antioxidant properties. The closeness of the relationship depended on the sample set; when all tested samples were included in correlation rows, poor associations were determined for both variable pairs TFC and DPPH, as well as for TFC and FIC. This may be a result of the* Astragalus *species belonging to different taxonomic units in the genus and, therefore, possessing different compositions of antioxidant agents. The different trends in correlation strength for pairs TFC versus DPPH and TFC versus FIC, which were apparent in the sample sets of the separate species, support the view that flavonoids from* A. glycyphyllos* extracts are more closely associated with scavenging of DPPH free radicals, while those from* A. cicer* have a closer association with FIC.

Isoflavone content was weakly correlated with antioxidant properties, TPC and TFC (data not shown). Statistical comparison of free-radical scavenging capabilities against FIC properties of the investigated materials showed a moderate but insignificant (*p* < 0.05) correlation. Results obtained in our study on the relationship between bioactive compounds and antioxidant properties are consistent with those of other researchers. Similarly, Tepavčević et al. [53] have documented that the DPPH scavenging activity correlated well with total polyphenolic content but did not correlate with total isoflavones in soybeans of different origin. Romani et al. [54] found no correlation between data on TPC and isoflavone content in isoflavone-based food supplements. Rau De Almeida Callou et al. [55] also reported the absence of a correlation between antioxidant capacity and isoflavone contents for soy beverages. Tusevski et al. [43] established a negative correlation between DPPH and FIC for Macedonian medicinal plants and proposed that this may be due to different reaction mechanisms involved in the two antioxidant determination methods. The antioxidant capacity of plant extracts is considered to depend on the specific combination of bioactive compounds and their synergistic interactions [56], or their additive or antagonistic responses [57].

In summary, our findings on the distribution of minerals and phenolic compounds, as well as the antioxidant properties, in samples from two* Astragalus *species from a temperate region have demonstrated the potential pharmaceutical and nutraceutical significance of these plants.

## 4. Conclusion

The study assuredly demonstrates the potential of plant material from* A. glycyphyllos* and* A. cicer* as a valuable source of iron and phenolic substances, including isoflavones, free-radical scavengers, and Fe^2+^ chelators. However, mineral and phytochemical compositions, as well as antioxidant properties, were found to be species-, plant growth stage-, and plant part-dependent features. The investigated plant material may be considered as a potential source of dietary supplements and pharmaceutical and nutraceutical products, depending on species, growth stage, and plant part.

## Figures and Tables

**Table 1:** Mineral profile of *A. glycyphyllos* and *A. cicer* plant material; whole aerial part (WP) of plants harvested at the vegetative stage (VS) and fully flowering stage (FS) and the separate parts of fully flowering plants (mean ± standard deviation).

Mineral	WP VS	WP FS	Stems	Leaves	Flowers
*A. glycyphyllos*					
Ash, g/100 g	10.82 ± 0.396	7.66 ± 0.007	4.11 ± 0.014	8.97 ± 0.233	7.88 ± 0.375
K, g/100 g	2.78 ± 0.060	2.18 ± 0.092	1.78 ± 0.115	2.22 ± 0.042	2.94 ± 0.045
Ca, g/100 g	1.55 ± 0.141	1.10 ± 0.042	0.701 ± 0.021	1.91 ± 0.113	0.463 ± 0.008
Mg, g/100 g	0.531 ± 0.008	0.511 ± 0.001	0.272 ± 0.003	0.596 ± 0.011	0.341 ± 0.052
P, g/100 g	0.320 ± 0.010	0.276 ± 0.017	0.198 ± 0.003	0.256 ± 0.003	0.428 ± 0.016
Zn, mg/100 g	2.44 ± 0.045	3.47 ± 0.113	2.30 ± 0.113	2.70 ± 0.141	4.76 ± 0.042
Fe, mg/100 g	65.29 ± 3.125	21.72 ± 1.131	15.85 ± 0.495	22.66 ± 1.670	14.10 ± 0.794

*A. cicer*					
Ash, g/100 g	11.23 ± 0.240	8.08 ± 0.014	4.23 ± 0.021	9.81 ± 0.113	9.18 ± 0.057
K, g/100 g	3.06 ± 0.080	1.99 ± 0.071	1.59 ± 0.023	2.38 ± 0.022	3.02 ± 0.099
Ca, g/100 g	1.60 ± 0.085	1.55 ± 0.044	0.649 ± 0.023	2.09 ± 0.085	0.643 ± 0.052
Mg, g/100 g	0.506 ± 0.006	0.538 ± 0.011	0.370 ± 0.008	0.545 ± 0.010	0.328 ± 0.023
P, g/100 g	0.366 ± 0.007	0.275 ± 0.016	0.180 ± 0.007	0.288 ± 0.020	0.473 ± 0.01
Zn, mg/100 g	2.79 ± 0.127	2.97 ± 0.113	1.88 ± 0.085	2.69 ± 0.085	4.61 ± 0.170
Fe, mg/100 g	30.19 ± 2.100	14.94 ± 0.764	6.78 ± 0.368	14.52 ± 0.651	13.13 ± 0.289

**Table 2:** Concentration of isoflavones (mg/100 g) in whole aerial part (WP) of plants harvested at the vegetative stage (VS) and fully flowering stage (FS) and the separate parts of fully flowering plants of *A. glycyphyllos* and *A. cicer*.

Isoflavone	Plant material				
	WP VS	WP FS	Stems	Leaves	Flowers
*A. glycyphyllos*					
Formononetin	9.24 ± 0.7^a^	5.50 ± 0.6	6.14 ± 0.6	5.05 ± 0.5	2.20 ± 0.3
Biochanin A	8.81 ± 0.8	3.17 ± 0.4	2.50 ± 0.3	4.43 ± 0.5	2.09 ± 0.2
Daidzein	<LOQ^b^	<LOQ	<LOQ	<LOQ	<LOQ
Genistein	2.23 ± 0.3	5.71 ± 0.6	7.29 ± 0.6	5.15 ± 0.5	4.83 ± 0.5
Sum	20.28	14.38	15.93	14.63	9.11

*A. cicer*					
Formononetin	10.85 ± 0.8	4.17 ± 0.4	5.55 ± 0.5	3.91 ± 0.4	1.30 ± 0.2
Biochanin A	11.40 ± 0.8	2.82 ± 0.3	3.14 ± 0.4	4.01 ± 0.5	2.49 ± 0.3
Daidzein	<LOQ	<LOQ	<LOQ	<LOQ	<LOQ
Genistein	<LOQ	4.21 ± 0.4	4.08 ± 0.4	4.33 ± 0.4	1.95 ± 0.2
Sum	22.25	11.20	12.77	12.25	5.74

^a^Mean of repetitions ± standard deviation; ^b^<LOQ: below the limit of quantification.

**Table 3:** Coefficients of linear correlation between the values of bioactive properties of the *Astragalus *plant material studied.

Species	*Agly* + *Acic*^a^	*Agly* ^b^	*Acic* ^c^	*Agly* + *Acic*	*Agly*	*Acic*	*Agly* + *Acic*	*Agly*	*Acic*
Character	TPC			TFC			DPPH		
TFC	0.855^*∗∗*^	0.969^*∗∗*^	0.947^*∗*^						
DPPH	0.315	0.952^*∗*^	0.977^*∗∗*^	−0.185	0.920^*∗*^	0.866			
FIC	0.739^*∗*^	0.872	0.861	0.450	0.873	0.973^*∗∗*^	0.574	0.852	0.520

Correlation coefficients (*r*) were computed for sample sets of ^a^*A*.  *glycyphyllos* with *A*. *cicer*, ^b^*A*.  *glycyphyllos*, and ^c^*A*.  *cicer* separately. ^*∗∗*, *∗*^Correlation is significant at *p* < 0.01 and *p* < 0.05 level, respectively.

## Supplementary Materials

Supplementary Materials The Supplementary Material represents a graphical abstract of the current study. It visually demonstrates the design of plant material of the two* Astragalus* species investigated and provides characters and properties tested.
