# Supplementary material for: Mineral and Phytochemical Profiles and Antioxidant Activity of Herbal Material from Two Temperate Astragalus Species
Source: Biomed Res Int. 2018 Jan 21;2018:6318630. doi: 10.1155/2018/6318630 (PMC5822765; doi:10.1155/2018/6318630)
Supplement: Supplementary Materials — The Supplementary Material represents a graphical abstract of the current study. It visually demonstrates the design of plant material of the two Astragalus species investigated and provides characters and properties tested. [file 6318630.f1.docx]

**Supplementary Material**

**Mineral and phytochemical profiles and antioxidant activity of herbal material from two temperate *Astragalus* species**

Bronislava Butkutė ^a^, Audronė Dagilytė ^b^, Raimondas Benetis ^b^, Audrius Padarauskas ^c^, Jurgita Cesevičienė ^a^, Vilma Olšauskaitė ^c^, Nijolė Lemežienė ^d^

^a^ Chemical Research Laboratory, Institute of Agriculture, Lithuanian Research Centre for Agriculture and Forestry, Instituto al. 1, 58344 Akademija, Kėdainiai distr., Lithuania

^b^ Department of Drug Chemistry, Faculty of Pharmacy, Lithuanian University of Health Sciences, A. Mickevičiaus 9, 44307 Kaunas, Lithuania

^c^ Department of Analytical and Environmental Chemistry, Vilnius University, Naugarduko 24, 03225 Vilnius, Lithuania

^d^ Department of Grass Breeding, Institute of Agriculture, Lithuanian Research Centre for Agriculture and Forestry, Instituto al. 1, 58344 Akademija, Kėdainiai distr., Lithuania

Correspondence should be addressed to Bronislava Butkutė; brone@lzi.lt


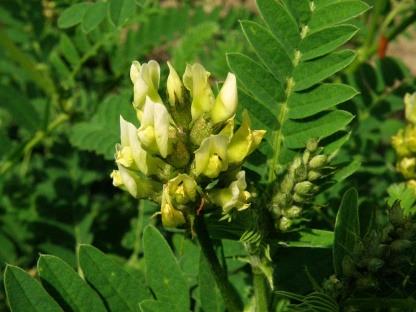

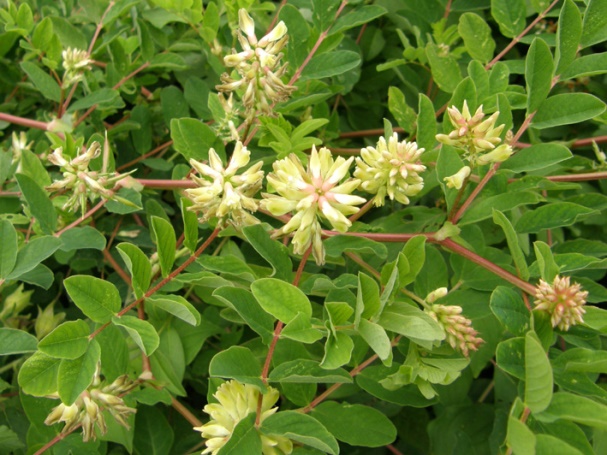

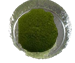


Swainsonine

Whole aerial plant part

***Astragalus cicer***

Isoflavones: formononetin, biochanin A, genistein, daidzein

Fe^2+^ chelating capacity

**Ash;**

Macroelements:

K, Ca, Mg, P;

Microelements:

Fe, Zn

Total phenolics

Total flavonoids

DPPH scavenging activity

(No ŽI-71)

***A. glycyphyllos*** (No ŽI-13)

Flowers

Leaves

Stems

Whole aerial plant part

Vegetative stage

Full flowering stage
